# Different causes and diverse outcomes of extremely rare septic cavernous sinus thrombosis complicated with internal carotid artery stenosis

**DOI:** 10.1186/s40001-021-00588-6

**Published:** 2021-10-06

**Authors:** Bo-An Chen, Zhuo-Hao Liu, Chi-Cheng Chuang, Cheng-Chi Lee

**Affiliations:** 1 Department of Neurosurgery, Linkou and Chang Gung University, Taoyuan, Taiwan, ROC; 2 Department of Neurosurgery, Chang Gung Memorial Hospital, Chang Gung University, 5 Fu-Shin Street, Gui-Shan Dist., Linkou, Taoyuan, Taiwan, ROC

**Keywords:** Septic cavernous sinus thrombosis, ICA stenosis, Endoscopic endonasal approach, Outcome

## Abstract

**Background:**

Cases of acute sphenoid sinusitis complicated by septic cavernous sinus (CS) thrombosis and internal carotid artery (ICA) stenosis are rarely reported. Different causative pathogens have been reported for this condition. We present two extremely rare and special cases with diverse clinical presentations and outcomes. Case 1 involved a female patient with less extensive sinusitis, but critical ICA occlusion. Case 2 involved a male patient with extensive pansinusitis, meningitis, cerebritis, and vasculitis due to fungal infection, but less stenosis of the ICA lumen. Both patients underwent surgical debridement and received broad-spectrum antibiotics. Additional anti-fungal medication was also administered in Case 2. However, outcomes differed considerably between cases.

**Discussion:**

Case 1 recovered with minimal neurological deficits and had Glasgow Outcome Scale (GOS) and modified Rankin Scale (mRS) scores of 5 and 2, respectively; however, the Case 2 had GOS and mRS scores of 3 and 4, respectively. Although rare, septic CS thrombosis with ICA stenosis can lead to unexpected and severe neurological sequelae. Fungal infection can result in catastrophic complications and poorer prognosis.

**Conclusion:**

In addition to early detection, aggressive surgical debridement and adequate antimicrobial treatment are crucial to satisfactory outcomes in patients with septic CS thrombosis complicated with ICA stenosis.

## Introduction

Septic cavernous sinus (CS) thrombosis is a rare but fatal complication of paranasal sinusitis [1, 15, 17]. In the pre-antibiotics era, this condition had high mortality (80%) and morbidity (75%) rates [20], while in the modern era these rates have decreased dramatically, but still remain relatively high. In-hospital case-fatality rates of up to 44.4% have been reported for septic CS thrombosis in immunocompromised patients and the rate of residual neurological deficits has been reported to be > 30% [4, 19]. CS thrombosis with internal carotid artery (ICA) stenosis or occlusion is rarer and may result in severe neurological deficits and poor outcomes. Here, we present two cases of septic CS thrombosis with ICA stenosis and the resultant neurological complications.

## Case presentation

### Case 1

**Fig. 1:**
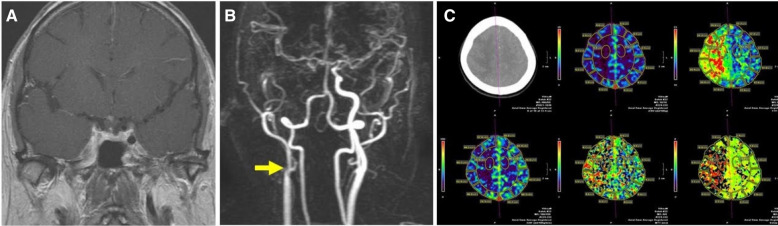
**A** Pre-operative brain magnetic resonance imaging showing right side cavernous sinus thrombosis with internal carotid artery occlusion. **B** Magnetic resonance angiography demonstrating retrograde occlusion at the common carotid bifurcation (*arrow*). **C** Computed tomography perfusion showing impaired perfusion in the right middle cerebral artery territory

A 60-year-old female presented with headache, fever, right ptosis, extraocular movement (EOM) limitation, diplopia, and left limb weakness after undergoing an odontogenic procedure and was admitted to a local hospital. Her left upper extremity was weaker (grade 2) than her lower extremity (grade 4); she had elevated C-reactive protein (CRP) levels (18.7 mg/L) and a high leukocyte count (19,300/μL). Brain magnetic resonance imaging (MRI) (Fig. 1A) revealed acute sphenoid sinusitis complicated by right side CS thrombosis with ICA wall thickening. Magnetic resonance angiography (MRA) revealed retrograde occlusion of the proximal cervical ICA segment and common carotid bifurcation (Fig. 1B, *arrow*). Although collateral circulation, mainly from the anterior communicating artery (AcomA), to the middle cerebral artery (MCA) territory was observed, it was insufficient; acute infarction was evident on the diffusion-weighted imaging (DWI) sequence (not shown). Patient symptoms did not resolve and worsened despite antibiotic treatment, ultimately resulting in transfer to our hospital. Debridement and drainage using the endonasal endoscopic approach (EEA) were promptly performed to reduce microbial load and to obtain cultures. Broad-spectrum antibiotics were administered based on culture results. The patient gradually recovered with improving mild left-sided hemiparesis and paresthesia. Causative pathogens were *Aggregatibacter aphrophilus, Streptococcus constellatus,* and *Veillonella* species. The post-operative course was uneventful and her neurological deficits improved gradually. Post-operative digital subtraction angiography (DSA) revealed total occlusion proximal to carotid bifurcation in the right ICA, collateral flow from the AcomA, and posterior circulation (not shown). Post-operative MRI (not shown) showed resolution of CS thrombosis, but the ICA total occlusion and infarction status remained unchanged. Computed tomography perfusion (CTP) revealed impaired perfusion in the right hemisphere, predominantly in the MCA territory (Fig. 1C). We did not prescribe anticoagulants and rehabilitation programs were initiated after her neurological status stabilized. Serial follow-up MRIs revealed unchanged ICA total occlusion and post-ischemic status. Finally, she recovered with minimal neurological deficits of left side hemiparesis and paresthesia; she had Glasgow Outcome Scale (GOS) and modified Rankin Scale (mRS) scores of 5 and 2, respectively. Extracranial–intracranial (EC–IC) bypass for salvageable resuscitation of the inadequately perfused right MCA territory was explained and suggested to the patient and her family; however, they refused this option.

### Case 2

**Fig. 2:**
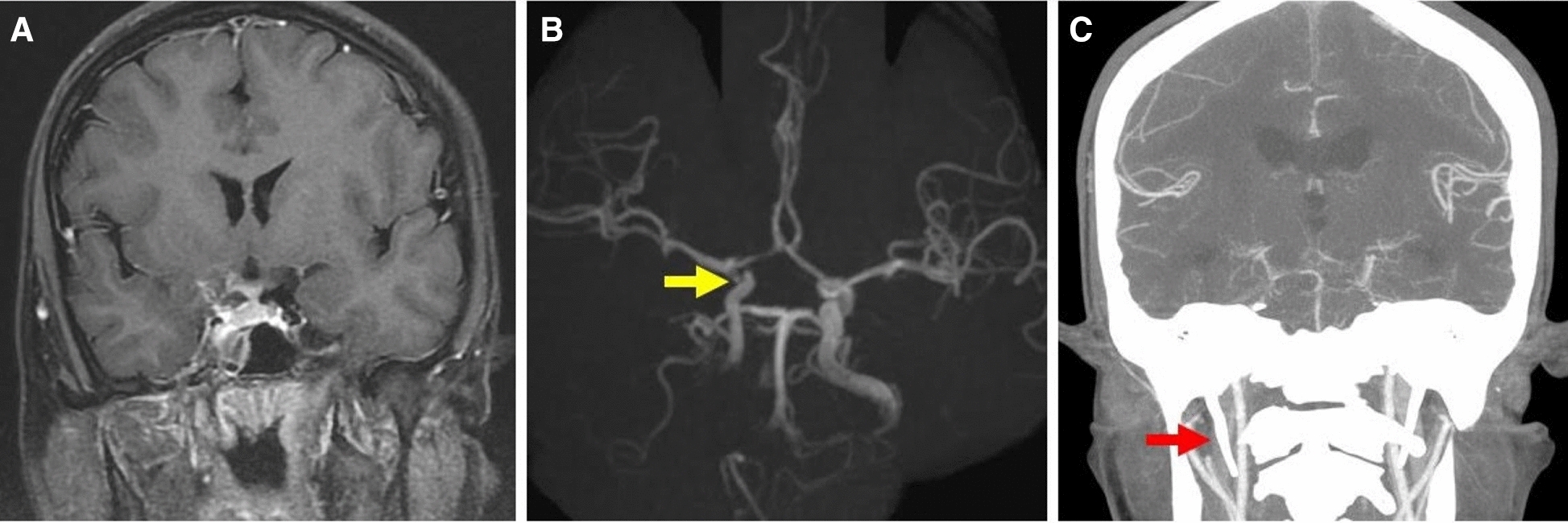
**A** Brain magnetic resonance imaging showing acute sphenoid sinusitis with right side cavernous sinus thrombosis and internal carotid artery stenosis. **B** Pre-operative magnetic resonance angiography demonstrating right internal carotid artery stenosis (*arrow*). **C** Post-operative computed tomography angiography showing reduced flow and a thrombus in the right internal jugular vein (*arrow*)

A 24-year-old otherwise healthy male with multiple teeth cavities and poor oral hygiene presented with headache and vomiting and developed an ill-looking appearance 1 week after undergoing functional endoscopic sinus surgery (FESS) for complicated sinusitis. The patient was referred to the outside emergency department (ED) where, upon physical examination, revealed febrile episodes, confused consciousness, and positive meningeal signs. MRI revealed acute sphenoid sinusitis complicated with right side CS thrombosis (Fig. 2A) and ICA stenosis (Fig. 2B, *arrow*). Acute ischemic changes at the basal ganglion were also observed upon DWI. Broad-spectrum empiric antibiotics were initially prescribed at the ED; however, progressively rising CRP levels (77.7 mg/L) and high leukocyte counts (17,500/μL) were detected. Therefore, EEA for debridement and drainage was promptly performed at our hospital to reduce microbial load and obtain cultures. Yellow purulent discharge was encountered intraoperatively after entering the sphenoid sinus. Unfortunately, the patient did not recover as expected immediately after the surgery. We performed computed tomography angiography (CTA) to exclude any possible progressive vascular complications. CTA revealed decreased cerebral flow, mainly in the MCA territory, and hydrocephalus caused by meningitis, and ventriculitis (not shown). Additionally, reduced flow in the internal jugular vein (IJV) suggested IJV thrombosis (Fig. 2C). The pathogens identified were *Propionibacterium avidum, P. acnes, Candida albicans, Cladosporium* species, and *Phoma* species. Anti-fungal medication was administered in addition to broad-spectrum antibiotics. Expected clinical improvement was not observed despite antimicrobial treatment at 3 weeks post-surgery. Follow-up MRI showed bilateral multiple infarctions in the basal ganglion and right MCA territory (not shown). Aggressive surgical intervention was not considered, and only extraventricular drainage (EVD) was performed for hydrocephalus. Anticoagulants were not prescribed because it was possible that repeated EVD operations would be needed until stabilization of central nervous system (CNS) infection. The patient’s condition improved after long-term administration of anti-fungal drugs. In this relatively stable condition, EC–IC bypass for salvageable resuscitation of the inadequately perfused right MCA territory and prevention of further extensive ischemic events was explained and suggested to the family; however, they refused this option. Finally, he recovered with moderate neurological deficits and fluctuating consciousness with GOS and mRS scores of 3 and 4, respectively.

## Discussion

Septic CS thrombosis caused by sphenoid sinusitis with severe complication of ICA stenosis or occlusion is very rare and results in catastrophic and irreversible sequelae. Only a few case reports have been published describing this condition; however, none of them reported two cases with different uncommon and atypical causative microorganisms, with diverse outcomes.

### Presentation

The most common presentations include febrile episodes, severe headache, eye swelling and redness, ptosis, exophthalmos, EOM limitation, and sometimes dysarthria or hemiparesis [4–8]. Case 1 presented with typical ptosis, EOM limitation, and hemiparesis, whereas Case 2 showed severe increased intracranial pressure (IICP), meningeal symptoms, and progression to disturbed consciousness. This severe clinical scenario might have resulted from fungal infection that is notorious for its rapid intracranial spread and tissue destruction, as demonstrated in our patient, despite prompt surgical intervention and antimicrobial treatment.

### Diagnosis

The initial clinical signs are so subtle that misdiagnosis of such a rare disease is commonplace; thus, imaging results are essential for confirming a diagnosis. CT is the first choice to evaluate the involved area, sinusitis severity, associated orbital tissue swelling, ophthalmic vein engorgement, and to exclude intracranial hemorrhage or infarction. However, MRI is a preferable diagnostic tool and can provide more detailed information than CT. With gadolinium enhancement, the cavernous sinus is well-enhanced; therefore, flow-void filling defects, indicative of CS thrombosis, can be delineated. Structures of and interactions between the sellar region, ICA contour, stenosis, and occlusion are also better visualized on contrast-MRI and MRA. DSA is not regarded as a routine examination or at an early stage, unless the patient is relatively stable, or EC–IC bypass has been considered for future revascularization. We did not perform routine cerebrospinal fluid (CSF) examinations by lumbar puncture, unless the patient had undergone EVD and the CSF samples could be withdrawn via EVD. This was because we feared microorganism dissemination from the infected sinus area to the relatively clean CNS space due to negative suction pressure after lumbar CSF tapping.

### Pathogens

The patients in our study were not infected by common typical species such as *Staphylococcus aureus, S. viridans, S. pneumoniae,* or other anaerobic bacteria. In Case 1, the final culture yielded *A. aphrophilus, S. constellatus,* and *Veillonella species*. *A. aphrophilus* has been reported to cause infective endocarditis and in a few cases, brain abscesses [13]. *S. constellatus* constitutes normal flora in the oral cavity, urogenital region, and intestinal tract; however, it may cause purulent infections in other organs. *S. constellatus* is a member of the *Streptococcus milleri group*, which have been commonly associated with abscesses, bacteremia, and subsequent septic shock [10] and have been reported to cause CS thrombosis [1, 15]. *Veillonella species* are anaerobic cocci that are frequently found in chronic periodontitis patients. They constitute normal flora of the oral ecosystem and interact with *Streptococci* [6, 11]. In Case 2, although the healthy immunocompetent male had poor oral hygiene, he was also infected with fungi. Fungal culture yielded *C. albicans, Phoma* species, and *Cladosporium* species. They all constitute normal flora, but have been reported to cause human infections in specific scenarios. *C. albicans* is a potentially infectious microorganism in immunocompromised patients. *Phoma* species are commonly observed in plants and soil and are rarely infectious in humans. *Cladosporium* species are commonly found in plants, and although rare, may be pathogenic to humans, causing lung and sinus infections. Additionally, Case 2 also showed evidence of IJV thrombosis on CTA; therefore, we considered his condition to be an atypical form of Lemierre’s syndrome [5, 14].

### Treatment

Antimicrobial medications, including empiric broad-spectrum antibiotics, should be initially prescribed and then adjusted according to culture results. Fungal infection should always be considered if the expected improvement is not observed. A literature review showed that only antibiotics were administered as primary therapy in reported cases of CS thrombosis [7, 8, 12, 19]; however, surgical drainage is strongly recommended (as in our cases) to reduce bacterial load and to prevent progressive vascular occlusion. EEA is the best approach because it is minimally invasive and less time-consuming. Corticosteroids may alleviate inflammatory damage and facilitate penetration of antibiotics through the blood–brain barrier [16]. Anticoagulant use remains controversial because it can reduce mortality in selected cases [2, 18], but increase the risk of intracerebral hemorrhage. We did not prescribe anticoagulants to our patients, especially Case 2, for fear of intracerebral hemorrhage due to the need of repeated EVD procedures. Furthermore, Joseph et al. [12] treated a patient with repeated endovascular angioplasties in the acute and subacute infectious stages that were complicated by iatrogenic dissection of the intrapetrous segment of the left ICA; these procedures did not have therapeutic benefits or result in clinical improvement. We do not recommend this endovascular procedure because the infected vessels are vulnerable to mechanical manipulation, and it could cause dissection or rupture, which should be avoided.

### CNS complications and outcomes

The most dangerous complications involve the CNS, these include meningitis [18], subdural abscess [3, 9], and stroke [9]. Although Case 1 had severe ICA occlusion with subsequent infarction in the MCA territory, she had better collateral circulation and neurological outcomes. Case 2 had less severe ICA stenosis with resultant multiple small infarcts, attributable to uncontrolled meningitis and vasculitis and had a poorer neurological outcome compared to Case 1. Therefore, since ICA was involved, complicated stenosis or occlusion could cause varied neurological deficits depending on the severity of acute sinusitis, degree of stenosis, collateral circulation status, and control of underlying infection. Although antibiotics were prescribed, and aggressive surgical drainage was performed, our patients showed vastly different outcomes indicating that even after prompt antibiotics and aggressive surgical treatments, irreversible dense neurologic deficits could occur, particularly in cases of fungal infections.

### Controversies

No consensus has been reached regarding antimicrobial treatment duration, anticoagulant use, and practicality of EC–IC bypass for such cases. Standard antimicrobial treatment duration has not been decided (range 2–8 weeks) [7, 12, 19]. Nevertheless, if the antimicrobial therapeutic course is not completed, symptoms and signs may recur after discharge; therefore, a complete treatment has been suggested, especially in cases highly suspected of CNS involvement. As previously stated, this might help in reducing mortality. However, anticoagulant use should be based on the patient’s condition and physicians’ expertise and requires close monitoring due to risk of intracerebral hemorrhage. Literature reports providing evidence and experience regarding the role of EC–IC bypass for ICA stenosis or occlusion caused by septic CS thrombosis are lacking. Endovascular interventions, such as balloon angioplasty and stent placement, are not appropriate due to complete occlusion or fragility of the infected ICA. In both stable and chronic stages, EC–IC bypass may be a feasible option for reestablishing adequate vascularization.

## Conclusions

Acute sphenoid sinusitis complicated with CS thrombosis and ICA stenosis is difficult to diagnose at an early stage. Prompt administration of empiric broad-spectrum antimicrobial agents and surgical drainage are critically important. Outcomes may differ owing to uncontrolled infections, particularly in cases of fungal infection. EC–IC bypass may be a reasonably suitable option when patients present in a stable condition.

## Data Availability

The authors confirm that the data supporting the findings of this study are available within the article [and/or] its additional materials.
